# Hydroxyzine Use in Preschool Children and Its Effect on Neurodevelopment: A Population-Based Longitudinal Study

**DOI:** 10.3389/fpsyt.2021.721875

**Published:** 2022-01-28

**Authors:** Hans J. Gober, Kathy H. Li, Kevin Yan, Anthony J. Bailey, Bruce C. Carleton

**Affiliations:** ^1^Division of Translational Therapeutics, Department of Pediatrics, Faculty of Medicine, University of British Columbia, Vancouver, BC, Canada; ^2^Department of Pharmacy, Kepler University Hospital, Linz, Austria; ^3^Therapeutic Evaluation Unit, Provincial Health Services Authority, Vancouver, BC, Canada; ^4^Pharmaceutical Outcomes Programme, BC Children's Hospital, Vancouver, BC, Canada; ^5^Department of Psychiatry, Faculty of Medicine, University of British Columbia, Vancouver, BC, Canada; ^6^BC Children's Hospital Research Institute, Vancouver, BC, Canada

**Keywords:** antihistamine, hydroxyzine, preschool-age children, atopic dermatitis, neurodevelopmental disorders, longitudinal study, tics, mental disorders

## Abstract

We identified the first-generation antihistamine hydroxyzine as the earliest and most frequently prescribed drug affecting the central nervous system in children under the age of 5 years in the province of British Columbia, Canada (1. 1% prevalence). Whereas, the antagonism of H1-receptors exerts anti-pruritic effects in atopic dermatitis and diaper rash, animal studies suggest an adverse association between reduced neurotransmission of histamine and psychomotor behavior. In order to investigate hydroxyzine safety, we characterized the longitudinal patterns of hydroxyzine use in children under the age of 5 years and determined mental- and psychomotor disorders up to the age of 10 years. We found significantly higher rates of ICD-9 and ICD-10 codes for disorders such as tics (307), anxiety (300) and disturbance of conduct (312) in frequent users of hydroxyzine. Specifically, repeat prescriptions of hydroxyzine compared to a single prescription show an increase in tic disorder, anxiety and disturbance of conduct by odds ratios of: 1.55 (95%CI: 1.23–1.96); 1.34 (95%CI: 1.05–1.70); and 1.34 (95%CI: 1.08–1.66) respectively in children up to the age of 10 years. Furthermore, a non-significant increased trend was found for ADHD (314) and disturbance of emotions (313). This is the first study reporting an association between long-term neurodevelopmental adverse effects and early use of hydroxyzine. Controlled studies are required in order to prove a causal relationship and to confirm the safety of hydroxyzine in the pediatric population. For the time being, we suggest the shortest possible duration for hydroxyzine use in preschool-age children.

## Introduction

Between birth and 5 years of age, the human brain exhibits its fastest rate of development (1). The use of drugs which affect the central nervous system (CNS) is challenging in children due to the lack of pediatric studies and the difficulty in diagnosis of mental disorders in preschool-aged children (2). The original purpose of our study was the assessment of psychotropic drug use and associated diagnosis patterns in children under the age of 5 years. We identified the first-generation antihistamine drug hydroxyzine, as the earliest prescribed drug affecting the CNS in infants and toddlers and even more so, as the most frequently prescribed sedating medication in children under the age of 5 years in British Columbia, Canada. In contrast to the second-generation antihistamines, exhibiting a high specificity for peripheral histamine H1-receptors, there is little knowledge on the complex neuro-pharmacodynamics and safety of the first-generation drugs especially in young children (3). Due to the increasing prevalence of allergic rhinitis in children of school age and in adolescents, studies were conducted to address the impact of disease and medication on learning and academic achievement. The result of clinical cohort studies in children at school age and adolescents with allergic rhinitis suggests that use of sedative antihistamine drugs has a more negative impact on learning and academic performance compared to allergic rhinitis alone or if non-sedative second-generation antihistamine drugs are used (4, 5).

However, the specific relevance of histamine neurotransmission in the brain has been addressed only so far in basic science and animal studies. Rat models have demonstrated the involvement of neuronal histamine in the formation of long-term memory, as early as in the 1980's (6). More recently, a mutation in histidine decarboxylase was identified to be associated with inherited Tourette's syndrome in men (7). Subsequent animal studies on mice deficient for histidine decarboxylase confirmed the phenotype of a tic spectrum disorder and the involvement of neuronal histamine on the regulation of psychomotor activity (8).

So far there is only one published controlled trial on the neuropsychiatric outcome of an antihistamine drug in children under the age of 5 years. This controlled prospective long-term safety study was conducted on cetirizine, an active metabolite of hydroxyzine. The trial enrolled 817 children between 1 and 2 years of age with atopic dermatitis treated with systemic cetirizine or placebo. No difference was found between cetirizine and placebo in cognitive abilities, behavior and development up to 18 months after discontinuation of therapy (9, 10). However, cetirizine is a second-generation antihistamine and minimally penetrates the blood brain barrier compared to its precursor molecule hydroxyzine (11). In contrast, a recent observational study provided evidence that early life exposure to antihistamine drugs, especially the first-generation drug diphenhydramine, may be an independent risk factor for development of attention-deficit hyperactivity disorder (ADHD) in childhood (12).

In Canada, hydroxyzine is licensed for the alleviation of pruritic skin disease, allergic rhinitis and for the mitigation of anxiety and nausea. The only pediatric safety study with hydroxyzine conducted in 1984 involved 12 children between 2 and 10 years of age with severe atopic dermatitis and a total drug exposure time of 2 weeks (13). This study focused on pharmacokinetics and dose finding and did not report any adverse effects besides sedation. There is no minimum age restriction for its use in pediatrics.

The lack of pediatric safety studies on hydroxyzine together with its frequent use in infants and toddlers, and animal studies suggesting a crucial function of histamine as neurotransmitter, prompted us to investigate hydroxyzine's safety.

The aims of the current study were to characterize the longitudinal patterns of systemic hydroxyzine prescription in children under the age of 5 years and to evaluate whether frequent use of hydroxyzine in this young population might be associated with mental- and psychomotor diseases.

## Methods

### Study Design and Data Sources

A population-based retrospective observational study was conducted using administration databases from the province of British Columbia, Canada. Data extracted includes all children under the age of 19 years who had prescription of drugs acting on the CNS between fiscal years 1997 and 2018. Research Ethics approval was provided by the University of British Columbia, Children's and Women's Hospital's Research Ethics Board (H18-01247).

The following health resource utilization data were obtained: Medical Services Plan (MSP) Payment Information File (14), Discharge Abstract Database (Hospital Separations) (15), PharmaNet Data and Consolidation File (MSP Registration & Premium Billing) (16). These data files provide patients demographics, diagnosis codes and prescription dispensing records. This study was focused on psychotropic drug use in children: 0–5 years of age.

### Hydroxyzine Administration in Children

Initial screening shows that among all CNS medications, hydroxyzine was the predominant one prescribed to children under the age of 5 years. For ease of administration to children, a liquid formulation of hydroxyzine is available in Canada (Atarax^®^ syrup). This sweet tasting syrup contains 473 ml with 0.95 gram of hydroxyzine and available only on prescription. In our data, the medication is mainly prescribed by one formula (HYDROXYZINE HCL 10MG/5ML oral solution).

### Diagnosis of Neurodevelopment Disorders

In the pediatric population, assessment of neurodevelopment disorders is a complex and challenging practice; there are fewer strict diagnosis tools like DSM-V used in adults. Our data only have the ICD-9 and ICD-10 codes available for the diagnoses of mental health conditions. Table 1 listed all ICD codes associated with mental- and psychomotor disorders according to their potential relevance in neurotransmission of histamine.

**Table 1:** ICD-9 and ICD-10 diagnosis codes for psychomotor and mental health disorders used in this analysis.

	**Disease**	**ICD-9 codes**	**ICD-10 codes**
Psychomotor disorders	Tic disorders	307	F95.0–F95.2 F95.8–F95.9
	Hyperkinetic syndrome of childhood	314	F90.0 F90.8–F90.9
Learning deficiencies	Intellectual disabilities	317–319	F70–F73 F78–F79
	Specific delays in development	315	F80.0–F80.2 F81.8–F81.9 F82 F84.1 F84.3–F84.5 F84.8–F84.9
Mental disorders associated with Tics	Anxiety disorders	300	F40.0–F40.2 F40.8–F40.9 F41.0–F41.3 F41.8–F41.9 F42.0–F42.2 F42.8–F42.9 F48.9 F93.0–F93.3 F99
	Disturbance of conduct	312	F91.0–F91.3 F91.8–F91.9 F92.0 F92.8–F92.9
	Disturbance of emotions	313	F93.8–F93.9 F94.0–F94.2 F94.8–F94.9 F98.8

We defined three disease categories for investigation: (1) psychomotor disorders; (2) learning deficiencies; and (3) mental disorders. Specifically, tic disorder and hyperkinetic syndrome in childhood were assigned to the psychomotor disorder; intellectual disabilities and specific delays in development were assigned to learning deficiencies; anxiety disorder, disturbance of conduct and disturbance of emotions were assigned to the mental disorders.

### Statistical Analysis

Our first analysis was generated for the distributions of patients under the age of 5 years across all the CNS drug classes. Hydroxyzine prescription patterns were followed for each patient from birth to age 5. Longitudinal patterns of use were compared in patients receiving only 1 prescription (short-term user), 2–4 prescriptions (intermediate user) and more than 4 prescriptions (long-term user). The frequency of specific mental and neuropsychiatric disorders was evaluated in those 3 groups by tracking the ICD-9 and ICD-10 diagnostic codes in two periods: birth to first dispensation, first dispensation to age 10. We conducted Cochran Armitage trend tests and logistic regression with generalized estimating equation (GEE) models to describe the prescription trends and their association with mental and neuropsychiatric disorders. The GEE logistic regression models were adjusted by patient's age, gender and geographic region of prescription. In a secondary analysis, we used Cox regression to model the incidence of tic development (time from initiation of hydroxyzine treatment to first tic diagnosis) and calculated the hazard ratios associated with cumulated hydroxyzine prescriptions. Statistical analyses were performed using SAS (version 9.4, SAS Inc, Cary NC).

## Results

### Hydroxyzine Prescription Patterns

**Figure 1:**
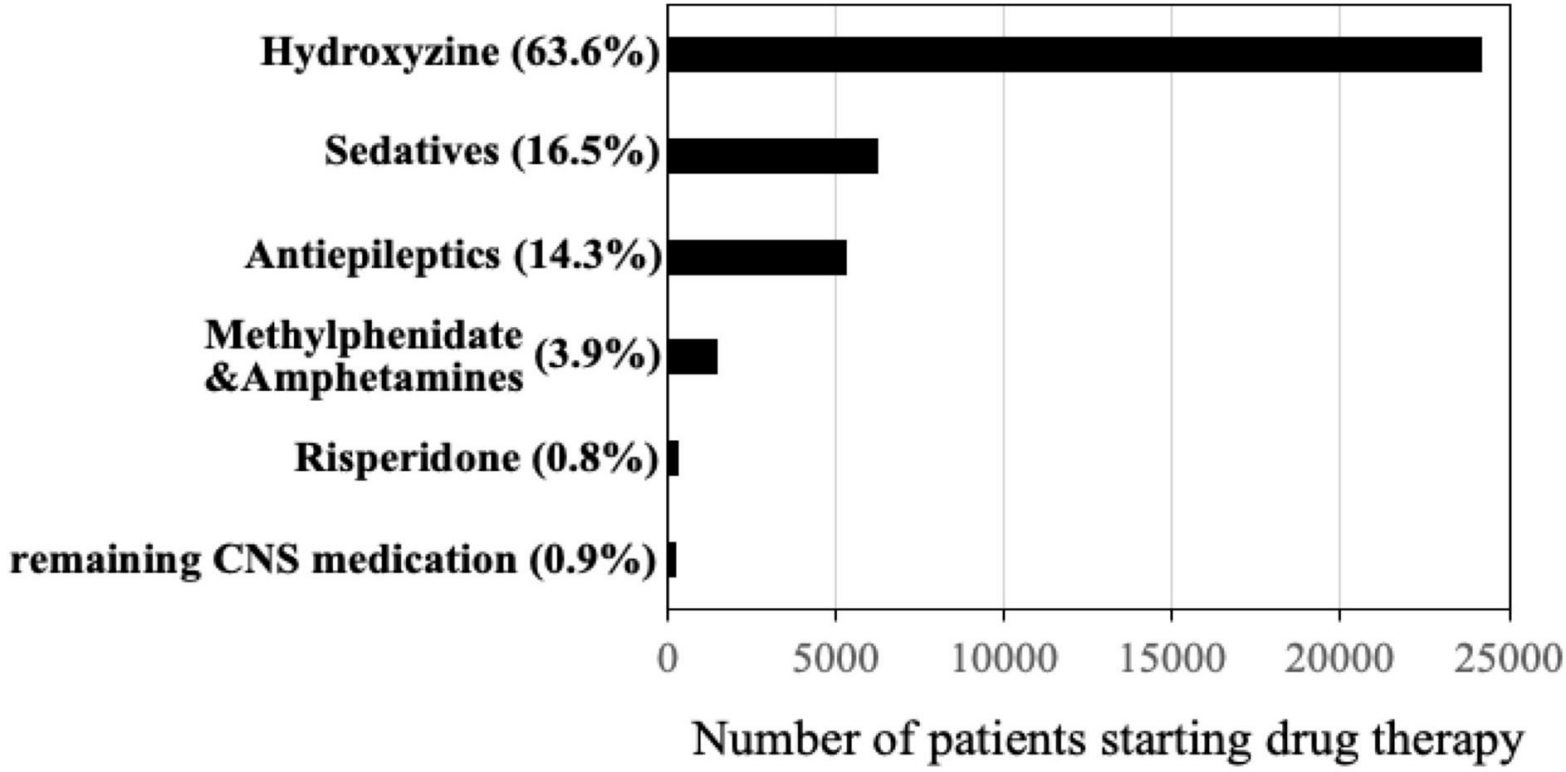
Distribution of first psychotropic drugs received under age of 5 years (total *n* = 38,016 children) in British Columbia, Canada (years 1997–2017).

**Figure 2:**
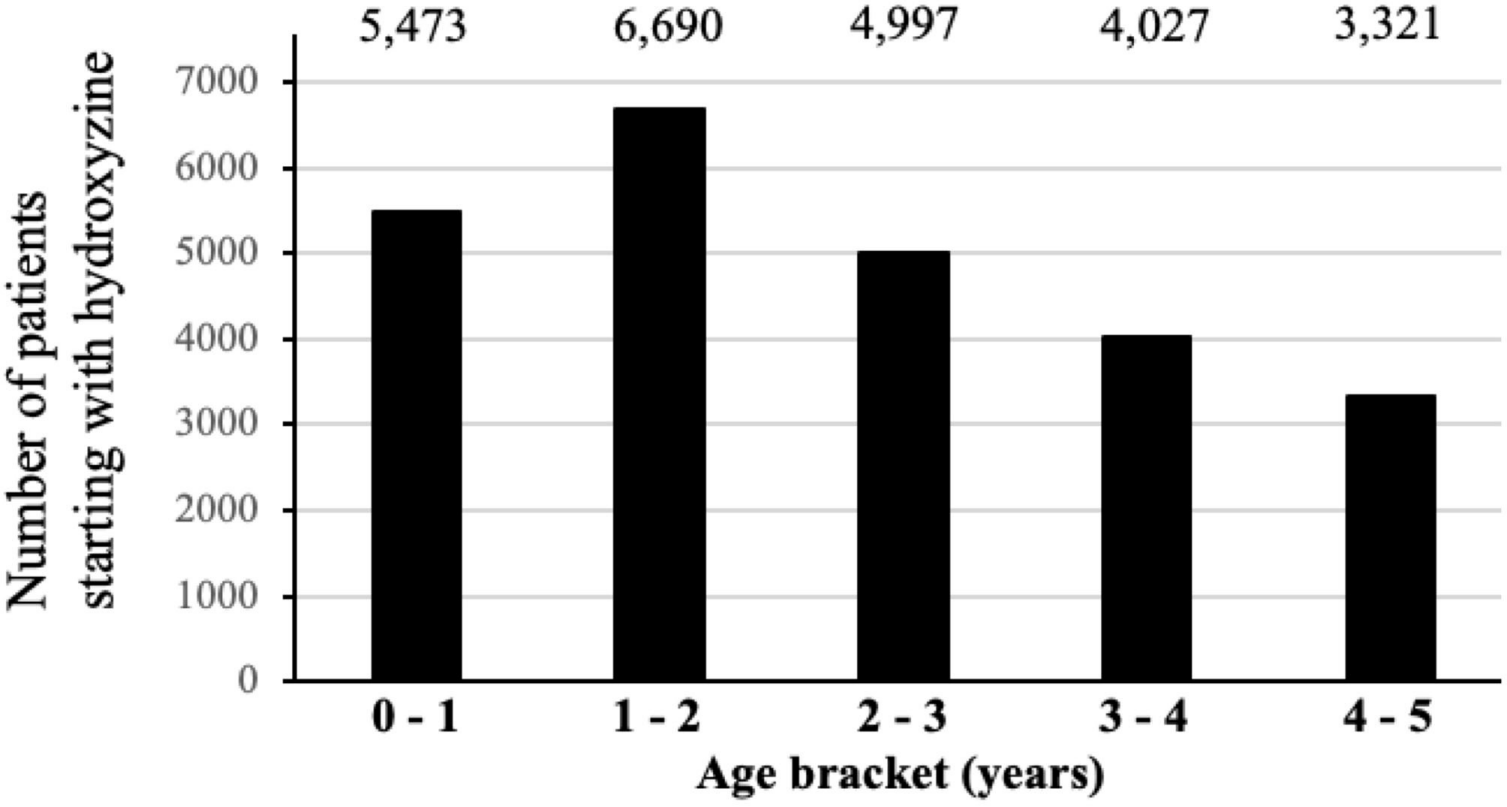
Distribution of children receiving hydroxyzine treatment by age level.

Figure 1 shows the distribution of first CNS medications used by children before age 5. Among a total of 24,371(63.6%) children prescribed hydroxyzine, 49.6% have received it before age 2 (Figure 2). The median age of starting hydroxyzine treatment was 2.2 years (IQR: 1.2–3.3). Hydroxyzine had been prescribed with a single formula (HYDROXYZINE HCL 10MG/5ML oral solution). This suggests that number of prescriptions up to age 5 can be used to represent cumulative exposure to the medicine. Within all the hydroxyzine users, 1,478 (6.1%) had received more than 4 repetitive prescriptions; 5,659 (23.2%) with 2–4 prescriptions; and 17,324 (70.7%) with one prescription only (Table 2). The group of frequent users (prescriptions ≥ 5) had an average of 9 prescriptions, corresponding to a maximum exposure of 8.5 grams before attaining school age.

**Table 2:** Frequencies of prescription and accumulative exposure to hydroxyzine in children under age of 5 years.

	**Short-term user** **1 prescription**	**Intermediate** **user 2–4** **prescriptions**	**Long-term** **user ≥ 5** **prescriptions**
Number of patients	17,234 (70.7%)	5,659 (23.2%)	1,478 (6.1%)
Number of prescriptions	17,234	14,148	13,302
(total)			
Average number of	1	2.5	9
prescriptions per			
patient			
Liters of hydroxyzine	0.47	1.17	4.23
syrup (average)			
Max. exposure to	0.95	2.4	8.5
hydroxyzine in grams			
(cumulative)			

### Indication for Prescribing

**Figure 3:**
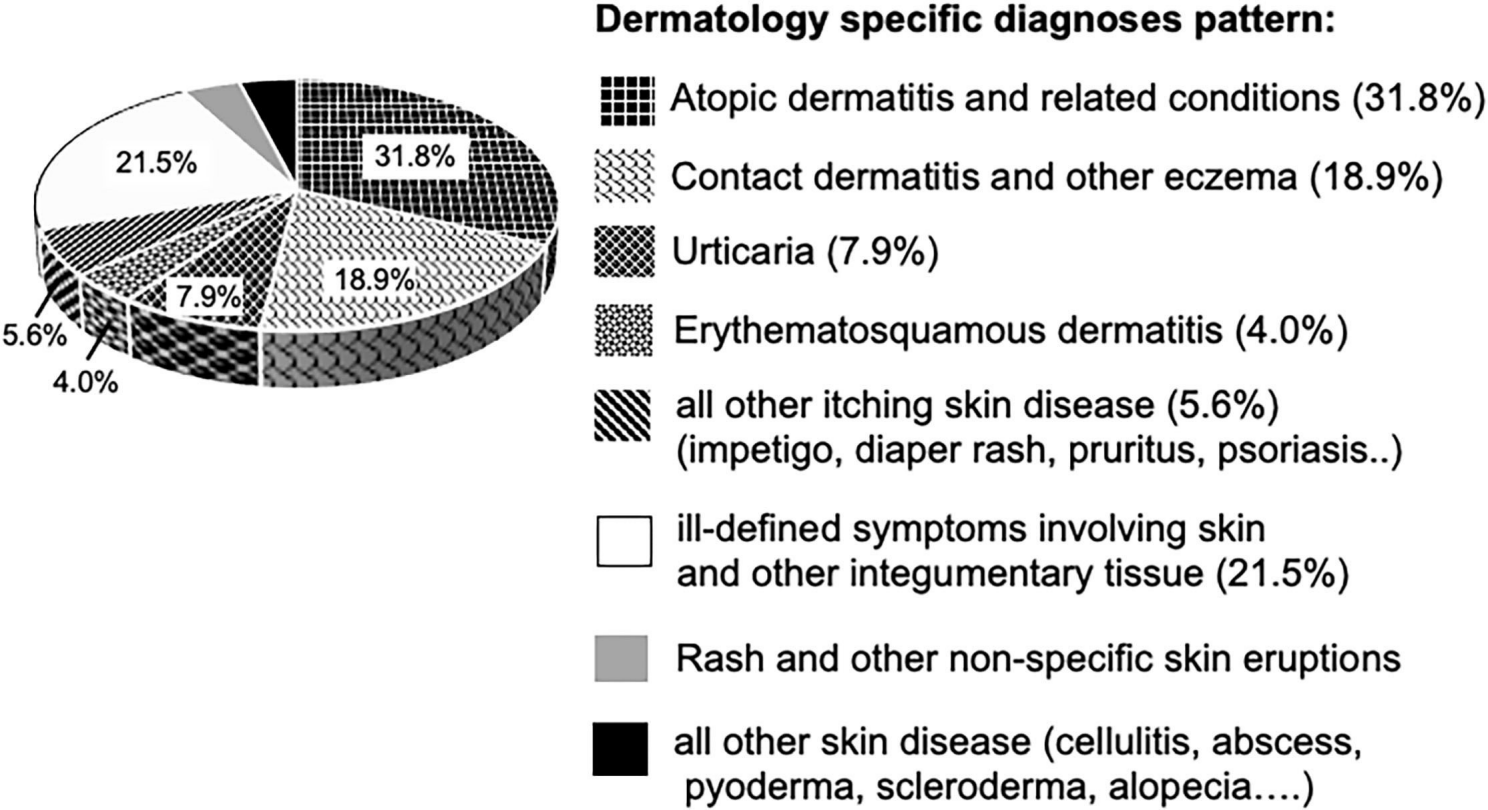
Dermatological disease diagnoses in hydroxyzine cohort (*n* = 20,226 children). Segments in diagram representing diagnoses associated with pruritus, are textured.

Among 24,371 hydroxyzine users, 20,226 (82.9%) had dermatological diagnoses ever before receiving hydroxyzine. By evaluation of the diagnosis codes related to dermatologic disease up to a period of 1 month prior to prescription, we found 68.2% of disorders be known to be associated with pruritus as predominant symptom, such as atopic- and contact dermatitis. 21.5% were ill-defined diagnoses of dermatologic conditions (ICD-9 code 782) and the remaining 10.3% relates to skin disease with an uncertain prevalence of pruritus, such as alopecia and rash diagnoses (Figure 3). In summary, the initial prescription of hydroxyzine in children under the age of 5 years was in accord with the licensed indication for pruritic skin conditions, with the obvious intention to alleviate the itch–scrape cycle for improving skin healing and nocturnal sleep.

### Neuropsychiatric Outcome Associated With Hydroxyzine Use

**Figure 4:**
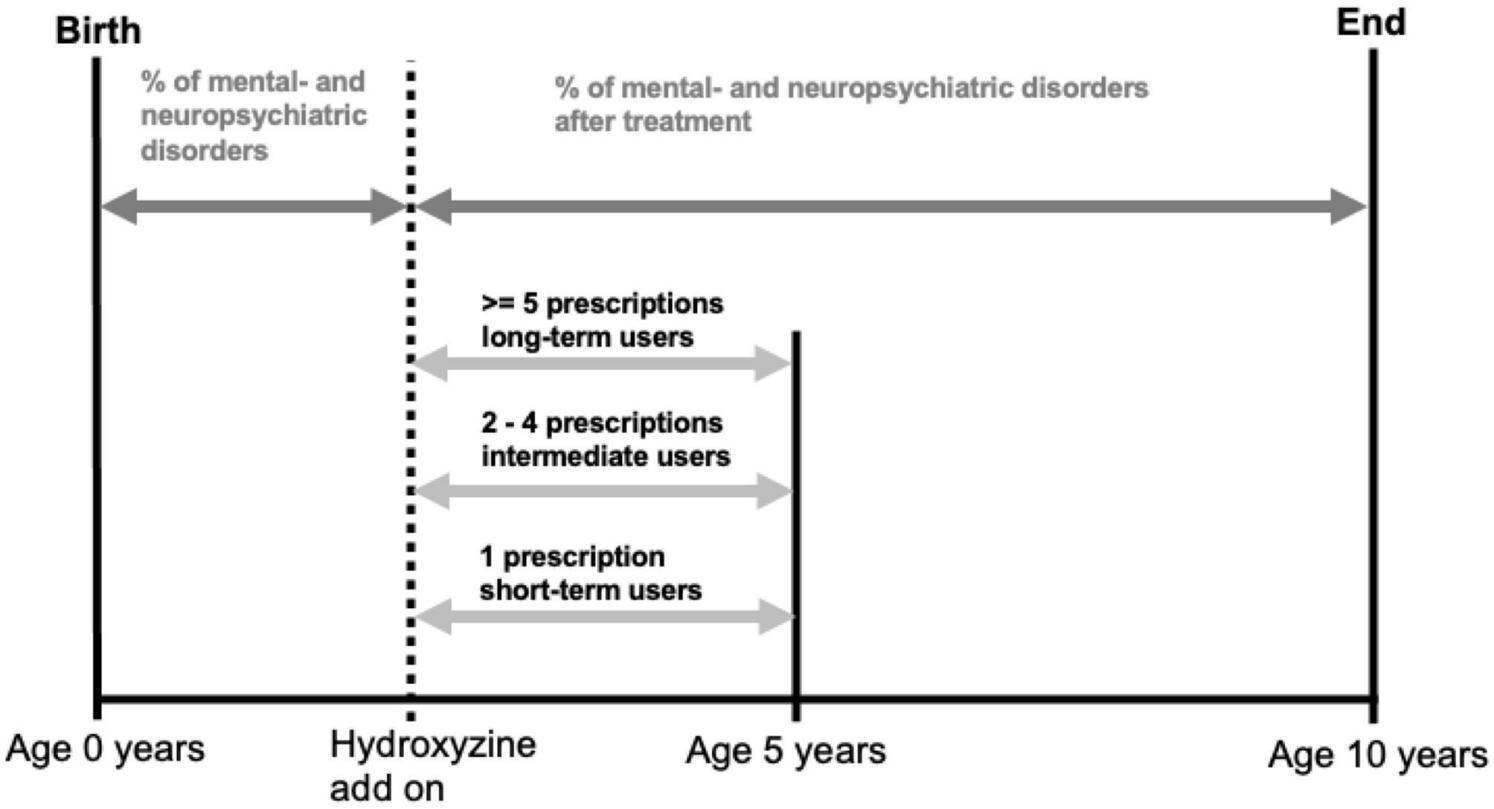
Illustration of quantifying hydroxyzine exposure and calculating prevalence of mental and neuropsychiatric disorders.

We used age of 5 as endpoint for the last refill of hydroxyzine in order to assess its effect on the most vulnerable phase of neurodevelopment, while age of 10 was chosen as endpoint for tracking disease development, due to the increased likelihood of symptom recognition and diagnosis at school age (Figure 4).

Before conducting the comparison in the rates of mental health disorders after receiving hydroxyzine in the three user's groups, we first examined if there were difference in preexisting conditions before treatment initiation. This baseline data showed no significant difference between the user's groups. As an example, tic was diagnosed overall by 1.92% in the whole cohort, with 1.98% in short-term users and 1.96% in frequent users (*p*-value = 0.448).

### Main Analysis

Table 3 shows the prevalence of mental health diagnoses for all children between initiation of hydroxyzine and age 10. Across the three levels of exposure to hydroxyzine, we found significant increasing trends of disorders in tic, anxiety and disturbance of conduct from short-term to long-term users. Specifically, the proportion of tic disorders goes from 3.77% in short-term user to 5.68% in frequent user; anxiety from 4.21 to 5.41%; and conduct disorder from 5.13 to 6.77%, respectively. After adjusting for age, gender, and health authority regions in multivariate logistic regression models, comparing frequent user of hydroxyzine to short-term user, the odds ratio for tic disorder is 1.44 (95% CI: 1.14–1.83), anxiety 1.28 (95% CI: 1.03–1.63) and disturbance of conduct 1.33 (95% CI: 1.07–1.66) respectively. ADHD and disturbance of emotions showed a tendency for increase with frequent use of hydroxyzine, while learning deficiencies were not altered by the quantity in use of hydroxyzine. Specifically, the proportion of ADHD goes from 6.21% in short-term user to 7.44% in frequent users (*p*-value = 0.164); and disturbance of emotions from 2.28 to 2.98% (*p*-value = 0.112) respectively.

**Table 3:** Prevalence of psychomotor and mental health disorders between initiation of hydroxyzine and age 10 years.

	**Short-term user**	**Intermediate user**	**Long-term user**	**Trend**
	***n* = 17,234**	***n* = 5,659**	***n* = 1,478**	**Test (*p*-value)**
Tic disorders	3.77 %	4.56 %	5.68 %	0.0002
Hyperkinetic syndrome of childhood	6.21 %	6.18 %	7.44 %	0.1649
Intellectual disabilities	0.35 %	0.46 %	0.34 %	0.4794
Specific delays in development	7.07 %	6.8 %	7.85 %	0.3734
Anxiety disorders	4.21 %	3.85 %	5.41 %	0.0286
Disturbance of conduct	5.13 %	5.48 %	6.77 %	0.0216
Disturbance of emotions	2.28 %	2.07 %	2.98 %	0.1121

### Sensitivity Analyses

We provide three additional analyses to assess the robustness of our results about the association between frequent hydroxyzine use and mental health disorders. Tic disorder is the primary outcome in these analyses.

### Children With Complete 10 Years Follow-Up Time

In this sub-analysis, we examined the children who had 10 full years of follow-up time (Table 4). This strategy allows equal time length for each child to assess their mental health conditions. Despite the reduction of number of patients to 18,758; tic disorders remained significantly higher in frequent users of hydroxyzine compared to short-term user (6.1 vs. 4.1%; odds ratio = 1.40; 95% CI: 1.08–1.81).

**Table 4:** Prevalence of psychomotor and mental health disorders between initiation of hydroxyzine and age 10 years (for children with full 10 years follow-up).

	**Short-term user**	**Intermediate user**	**Long-term user**	**Trend**
	***n* = 13,194**	***n* = 4,390**	***n* = 1,174**	**Test (*p*-value)**
Tic disorders	4.11 %	4.62 %	6.05 %	0.0046
Hyperkinetic syndrome of childhood	6.94 %	6.97 %	7.75 %	0.5796
Intellectual disabilities	0.34 %	0.48 %	0.43 %	0.4229
Specific delays in development	6.95 %	6.51 %	8.01 %	0.1928
Anxiety disorders	4.45 %	3.85 %	5.37 %	0.0541
Disturbance of conduct	6.06 %	6.08 %	7.58 %	0.111
Disturbance of emotions	2.53 %	2.26 %	3.41 %	0.0813

### Children With Dermatological Conditions

As our data only include children who received psychotropic medications; children who had dermatological diseases, but not using psychotropic drugs were not available. This makes it difficult to get a complete cohort of children with dermatological diseases. Nevertheless, we conducted the comparison among children with dermatologic conditions (20,226 out of the total 24,371). Tic disorders goes from 3.8% in short-term users to 5.3% in frequent users (odds ratio = 1.38; 95% CI: 1.08-1.80).

### Time From Hydroxyzine Initiation to Tic Development

In this analysis, we used Cox regression to model the time from initiation of hydroxyzine to first tic development. After receiving hydroxyzine, the median time of developing tic is 3.5 years (IQR: 1.5–5.6). By taking short-term users as the reference group, the hazard ratio for users with 2-4 prescriptions was 1.15 (95% CI: 0.99–1.34), and the hazard ratio for users with 5 or more prescriptions was 1.36 (95% CI: 1.07–1.73).

## Discussion

We found that the repetitive use of the first-generation antihistamine drug hydroxyzine in children of preschool age, was associated with elevated rates for tic disorder, anxiety and disorder of conduct up to the age of 10 years, by odds ratios of 1.55 (95%CI: 1.23–1.96); 1.34 (95%CI: 1.05–1.70) and 1.34 (95%CI: 1.08–1.66) respectively. Hereby, repetitive use was defined as a minimum of 5 prescriptions, which correspond to an accumulative exposure to hydroxyzine of more than 3.8 g before the age of 5 years. Review of the literature, such as PubMed, Micromedex, regulatory drug information for hydroxyzine syrup and the pharmacovigilance data analysis tool OpenVigil, did not reveal any information or surveillance data for tic disorders from the use of antihistamine medication. To our knowledge, there is no previous study reporting an association between extensive antihistamine drug use and the subsequent occurrence of tics, conduct- and anxiety disorders. Our investigation was originally driven on findings from using animal data (6, 8) and a human genetic linkage study (7), which demonstrate the involvement of histaminergic neurons in psychomotor behavior.

Study limitations are the absence of an untreated cohort and the lack of information regarding concomitant medication outside of CNS medication and OTC drugs. Therefore, we can't exclude a direct association between tic disorder and the severity of atopic dermatitis. There is evidence that atopic dermatitis is associated with the occurrence of cancer, cardiovascular- and neuropsychiatric disease (17). Although, a recent meta-analysis encompassing 35 studies found that children and adolescents with atopic dermatitis have an overall higher risk of total mental disorders, they did not detect a significant difference in any specific disease (18). Longitudinal studies adjusted for medication usage are missing to confirm a direct causal relationship between atopic dermatitis and neuropsychiatric disorders.

Another limitation in our study is the absence of information on OTC drug usage in Canada, such as diphenhydramine, since a recent publication has shown evidence for early life exposure to diphenhydramine as independent risk factor for the development of ADHD (12). Another limitation is the reliance on the rather general code 307 in the ICD-9 classification to capture tic disorders. The ICD-9 diagnostic codes specific for tic disorder are not billable by health insurances and therefore rarely used in British Columbia. This fact makes it also impossible to further distinguish between Tourette's syndrome (307.23), transient (307.21)- and chronic tic disorders (307.22).

The strengths of this study are firstly the longitudinal character, which allows a follow up of each individual patient from birth to the age of 10 years in drug usage and diagnosis. Second, the combination of drug usage with medical diagnoses, encompassing the entire spectrum of diagnostic codes in hospital- and ambulatory care settings of every patient from birth to the age of 10 years. Third, the length of the study period provides a sufficient number of patients to improve statistical power, even if subgroups are analyzed. Lastly, the previous published data on animal studies allowed a hypothesis driven investigation of adverse effects, further strengthening the outcome we found in our patient cohorts.

Tic is a neuropsychiatric disease frequently observed in children at school age with an average prevalence of 2.99% (95% CI: 1.60–5.61), largely dependent on age cohort and study conditions applied (19). Although tics may resolve without treatment in most patients, later recurrences in adolescence or adulthood and psychiatric comorbidities are characteristics of this disease. Approximately 85% of children with chronic tic disorder have an associated mental disease, such as anxiety, attention deficit hyperactivity disorder (ADHD), disorders of conduct, obsessive-compulsive disorder or disturbance of emotions (20, 21). Indeed, we could confirm elevations in diagnostic codes for ADHD and anxiety in our cohort of long-time hydroxyzine user. The typical onset of tics occurs between ages 3 and 8 years and greatest severity is reported by the age of 10 years (19). Although in many cases its manifestations largely remit by adulthood, the disorder can persist for life. In our study we have limited the follow up to the age of 10 years, since highest severity and subsequent diagnosis concurs with early school age. It remains to be investigated whether the remission of tics by adulthood, completely resolves the predisposition for the associated psychiatric diseases, such as anxiety and obsessive compulsive disorder.

Hydroxyzine is considered as a selective antagonist for the H1-receptor. While antagonism of H1-receptors by antihistamine drugs at the cerebral cortex and medulla oblongata are considered to be responsible for the sedating effects, the antagonism of histamine at the hippocampal-cortical circuit may interfere with memory formation (22). It remains to be elucidated whether the observed adverse effects on memory of those first-generation antihistamine drugs in elderly patients is mediated via their antagonism of cerebral acetylcholine receptors or rather histamine-receptor antagonism. However, blockade of the neuronal histaminergic innervation in the striatum, as part of the basal ganglia, is most likely responsible for the adverse effect on psychomotor behavior observed in Tourette patients with defect in histidine decarboxylase (7).

The increasing prevalence of atopic dermatitis in infants and toddlers is the driving force behind the frequent use of hydroxyzine and other antihistamines. Atopic dermatitis is associated with sleep disturbance in children due to pruritus (23, 24). Despite the recommendation to prefer second-generation antihistamine drugs, such as loratadine, for treatment of pruritic skin disease (25), the adverse effect of sedation in first-generation antihistamines may be considered as advantage in children with additional sleep problems. Although we were unable to find any evidence in our analysis, we cannot exclude that the prolonged prescription of hydroxyzine in our long-time user cohort is motivated due to the convenience of sedation. Besides hydroxyzine, diphenhydramine is available as sedative first-generation antihistamine in a liquid syrup formulation for infants and toddlers and sold as OTC drug Benadryl®. This drug was introduced in 1946 before current licensing standards, and thus it did not pass the rigorous safety and efficacy standards required today (26). Such as for hydroxyzine, dosing regimes and age limits are not precisely specified for diphenhydramine.

In summary, our study found an association between the prevalence of mental disorders and the frequency of hydroxyzine prescription in preschool-age children. Controlled studies are required to proof a causal relationship between frequency of hydroxyzine use and the incidence of tics and mental disorders. The safety of hydroxyzine needs to be reassessed and it should be provided for a limited duration only. In addition, alternative therapies for atopic dermatitis and nocturnal itching, such as local antihistamines or corticosteroids, should be considered in preschool-age children. If emphasis is placed on the treatment of sleep disorders, alternative sedatives with minimum disturbance of sleep architecture, such as liquid trazodone formulations may be considered for children with neurological disorders.

## Data Availability Statement

The raw data supporting the conclusions of this article will be made available by the authors, without undue reservation.

## Ethics Statement

Research Ethics Approval was provided by the University of British Columbia, Children's and Women's Hospital's Research Ethics Board (H18-01247). Written informed consent from the participants' legal guardian/next of kin was not required to participate in this study in accordance with the national legislation and the institutional requirements.

## Author Contributions

All authors listed have made a substantial, direct, and intellectual contribution to the work and approved it for publication.

## Funding

This study was funded by the Therapeutic Evaluation Unit of the British Columbia Provincial Health Services Authority.

## Author Disclaimer

All inferences, opinions, and conclusions drawn in this publication are those of the authors, and do not reflect the opinions or policies of the Data Steward(s).

## Conflict of Interest

The authors declare that the research was conducted in the absence of any commercial or financial relationships that could be construed as a potential conflict of interest.

## Publisher's Note

All claims expressed in this article are solely those of the authors and do not necessarily represent those of their affiliated organizations, or those of the publisher, the editors and the reviewers. Any product that may be evaluated in this article, or claim that may be made by its manufacturer, is not guaranteed or endorsed by the publisher.
