# Supplementary material for: Hydroxyzine Use in Preschool Children and Its Effect on Neurodevelopment: A Population-Based Longitudinal Study
Source: Front Psychiatry. 2022 Jan 28;12:721875. doi: 10.3389/fpsyt.2021.721875 (PMC8832122; doi:10.3389/fpsyt.2021.721875)
Supplement: Supplementary file 1 [file Table_1.DOCX]

**Supplementary Table**

Compilation of all medication acting on the central nervous system, prescribed to children under age of 5 years in British Columbia, Canada (years 1997 – 2017), and registered in the PharmaNet Database.

| **Benzodiazepines** | Alprazolam  Bromazepam  Clobazam  Clonazepam  Clorazepate  Diazepam | Lorazepam  Midazolam  Nitrazepam  Oxazepam  Temazepam  Triazolam |
| --- | --- | --- |
| **Sedatives**  besides Benzodiazepines | Chloral hydrate  Ethchlorvynol  Paraldehyde  Zolpidem  Zopiclone |  |
| **Antiepileptics**  besides Benzodiazepines | Brivaracetam  Carbamazepine  Divalproex  Ethosuximide  Fosphenytoin  Lacosamide  Lamotrigine  Levetiracetam | Oxcarbazepine  Phenytoin  Phenobarbital  Rufinamide  Stiripentol  Topiramate  Valproic acid  Vigabatrin |
| **Stimulant drugs** | Dextroamphetamine  Lisdexamfetamine  Methylphenidate |  |
| **Antipsychotic drugs** | Aripiprazole  Asenapine  Chlorpromazine  Droperidol  Haloperidol  Loxapine  Methotrimeprazine | Olanzapine  Pimozide  Pipotiazine palmitate  Quetiapine  Risperidone  Thioridazine |
| **Antidepressant drugs** | Amitriptyline  Bupropion  Citalopram  Clomipramine  Desipramine  Doxepin  Duloxetine  Escitalopram  Fluoxetine  Fluvoxamine | Imipramine  Mirtazapine  Nefazodone  Nortriptyline  Paroxetine  Sertraline  Trazodone  Trimipramine  Venlafaxine  Vortioxetine |
| **Diverse** | Almotriptan  Apraclonidine  Atomoxetine  Buspirone  Caffeine  Clonidine  Dexmedetomidine  Disulfiram  Eletriptan  Flunarizine  Gabapentin  Guanfacine  Hydroxyzine  Ketamine | Lithium  Magnesium sulfate  Modafinil  Naloxone  Naltrexone  Prazosin  Pregabalin  Rizatriptan  Sibutramine  Sumatriptan  Tetrabenazine  Tryptophan  Zolmitriptan |
